# Supplementary material for: Iterative improvement in the automatic modular design of robot swarms
Source: PeerJ Comput Sci. 2020 Dec 7;6:e322. doi: 10.7717/peerj-cs.322 (PMC7924708; doi:10.7717/peerj-cs.322)
Supplement: Supplemental Information 3 [file peerj-cs-06-322-s003.zip › argos3/doc/api/standalone/a00597_source.html]

ARGoS: plugins/simulator/media/led\_medium.h Source File


- Main Page
- Related Pages
- Namespaces
- Classes
- Files

- File List
- File Members

# plugins/simulator/media/led\_medium.h

Go to the documentation of this file.

```
00001 #ifndef LED_MEDIUM_H
00002 #define LED_MEDIUM_H
00003 
00004 namespace argos {
00005    class CLEDMedium;
00006    class CLEDEntity;
00007 }
00008 
00009 #include <argos3/core/simulator/medium/medium.h>
00010 #include <argos3/core/simulator/space/positional_indices/positional_index.h>
00011 #include <argos3/plugins/simulator/entities/led_entity.h>
00012 
00013 namespace argos {
00014 
00015    class CLEDMedium : public CMedium {
00016 
00017    public:
00018 
00022       CLEDMedium();
00023 
00027       virtual ~CLEDMedium();
00028 
00029       virtual void Init(TConfigurationNode& t_tree);
00030       virtual void PostSpaceInit();
00031       virtual void Reset();
00032       virtual void Destroy();
00033       virtual void Update();
00034 
00039       void AddEntity(CLEDEntity& c_entity);
00040 
00045       void RemoveEntity(CLEDEntity& c_entity);
00046 
00051       CPositionalIndex<CLEDEntity>& GetIndex() {
00052          return *m_pcLEDEntityIndex;
00053       }
00054 
00055    private:
00056 
00058       CPositionalIndex<CLEDEntity>* m_pcLEDEntityIndex;
00059 
00061       CLEDEntityGridUpdater* m_pcLEDEntityGridUpdateOperation;
00062 
00063    };
00064 
00065 }
00066 
00067 #endif
```

---

Generated on 10 Jul 2018 for ARGoS by 
 1.6.1
